# Abcès froid dorsolombaire révélant un mal de Pott

**DOI:** 10.11604/pamj.2017.27.2.11174

**Published:** 2017-05-02

**Authors:** Kouame Jean–Eric Kouassi, Loukou Blaise Yao, Bada Justin Léopold Niaore Sery, Kouamé Innocent M’bra, Koffi Léopold Krah, Grah Franck Lohourou, Michel Kodo

**Affiliations:** 1 Service d’Orthopédie, Traumatologie, CHU de Bouake, Côte d’Ivoire

**Keywords:** Abcès froid, mal de Pott, osteoarticulaire, spondylodiscite, tuberculose, Cold abscess, Pott’s disease, osteoarticular, spondylodiscitis, tuberculosis

## Abstract

Les abcès froids tuberculeux représentent une forme rare et inhabituelle de tuberculose extra pulmonaire. Nous rapportons un cas d’abcès froid dorsolombaire révélant un mal de Pott sans complications neurologiques chez un patient de 27 ans, présentant depuis 05 mois une tuméfaction dorsolombaire gauche. L’examen du prélèvement à l’issu d’une incision et drainage de l’abcès a permis de confirmer l’origine tuberculeuse. Un traitement antituberculeux de 12 mois permettait une guérison avec une séquelle à type de gibbosité modérée.

## Abstract (en)

Tuberculous cold abscesses are a rare and unusual form of extrapulmonary tuberculosis. We here report the case of a 27-year old patient with a 5-month history of left dorsolumbar swelling presenting with dorsolumbar cold abscess revealing Pott’s disease without neurological complications. Examination of pus sample collected at the time the abscess was incised and drained helped to confirm the diagnosis of cold abscesses of TB origin. The patient underwent 12 months of anti-tuberculosis treatment which lead to a faster recovery; the sequelae was slight gibbosity.

## Introduction

Le mal de Pott, ou spondylodiscite tuberculeuse est la forme la plus fréquente des tuberculoses ostéoarticulaires, elle s’étend très souvent dans les parties molles pré et latéro-vertébrales sous la forme d’abcès froid [1, 2]. Les abcès froids tuberculeux représentent une forme rare et inhabituelle de tuberculose extra pulmonaire. Ils représentent 1% des formes de tuberculose [3]. Nous rapportons un cas de mal de Pott découvert à l’occasion d’un volumineux abcès froid pottique sans trouble neurologique.

## Patient et observation

**Figure 1:**
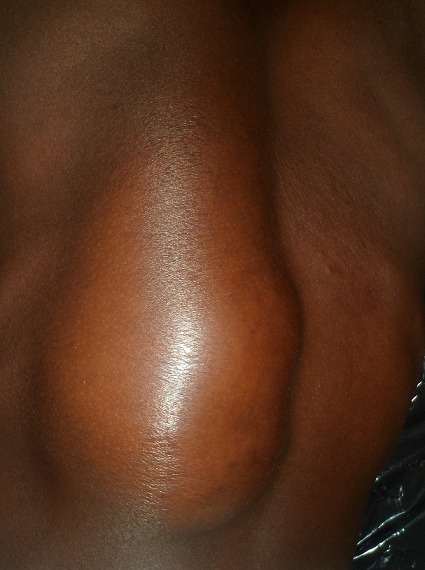
Abcès froid volumineux dorsolombaire, molle, indolore, non fistulisé

**Figure 2:**
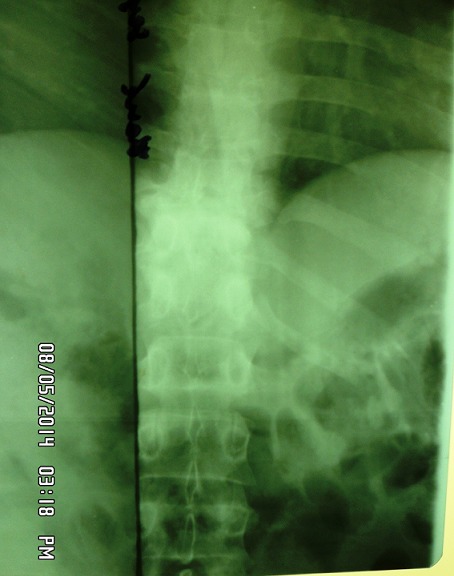
Radiographie du rachis dorsolombaire montrant des lésions de D10-D11 avec pincement discal, une érosion des plateaux vertébraux et une osteocondensation

**Figure 3:**
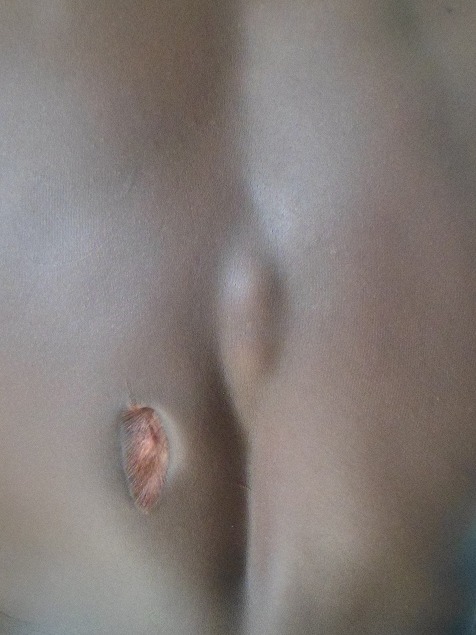
Disparition complète de la tuméfaction avec une gibbosité séquellaire après traitement

Un patient âgé de 27 ans avec une notion de contage tuberculeux, porteur d’une tuméfaction dorsolombaire gauche évoluant depuis 5 mois. Il a présenté une fièvre, un amaigrissement (avec une perte pondérale chiffrée à 18 kg), une asthénie et une anorexie. Il a consulté pour une dorsalgie en regard de la tuméfaction. L’examen clinique avait noté une température à 38°6C, un poids de 57kg pour une taille 175cm (indice de masse corporel 18,62 kg/cm^2^. L’examen physique a mis en évidence une volumineuse tuméfaction dorsolombaire gauche ovalaire mesurant 24cm de grand axe et 12cm de petit axe, de consistance molle, indolore, fluctuante, luisante non fistulisée (Figure 1), une douleur modérée à la palpation des épines dorsolombaires. L’examen pleuropulmonaire et neurologique était normal. La sérologie VIH était négative, la recherche de bacille de koch dans les crachats était négatif, l’intradermoréaction à la tuberculine était positif à 13 mm. La radiographie du rachis dorsolombaire a mis en évidence une lyse vertébrale intéressant la dixième et onzième vertèbre dorsale (D10-D11) avec pincement discal, une érosion des plateaux vertébraux et une ostéocondensation (Figure 2). L’échographie de la tuméfaction a mis en évidence la présence d’une collection liquidienne latéro-vertébrale infiltrant les muscles de voisinage. La radiographie du thoracique était normale. Le Scanner et l’IRM rachidien n’ont pu être réalisés. Il a été réalisé une incision drainage de la tuméfaction. L’analyse du prélèvement a montré la présence de bacille acido-alcooloresistant à l’examen direct et de Bacille de Koch à la culture. Un traitement antituberculeux a été entrepris sur une période de 12 mois, avec deux mois de phase d’attaque associant quatre antituberculeux majeurs: Rifampicine, Isoniazide, Ethambutol, Pyrazinamide, suivi d’une phase d’entretien associant Rifampicine et Isoniazide pendant dix mois. Au dernier recul de 2 ans, l’évolution a été favorable avec une disparition complète de l’abcès froid et une amélioration de l’état général du patient. Il avait une séquelle à type de gibbosité modérée (Figure 3).

## Discussion

Les abcès froids tuberculeux représentent une forme rare et inhabituelle de tuberculose extra pulmonaire. Ils représentent 1% des formes de tuberculose [1]. L’abcès froid pottique est le plus souvent observé dans le cadre des manifestations cliniques de la tuberculose vertébrale [4]. La spondylodiscite tuberculeuse ou mal de Pott, est l’atteinte de la colonne vertébrale incluant le sacrum et caractérisé par l’atteinte du disque intervertébral et des deux vertèbres adjacentes dans sa forme classique [4]. La colonne dorsolombaire est le siège le plus fréquemment atteint (95%), alors que le rachis cervical n’est touché que dans 5% des cas [2]. La tuberculose vertébrale s’étend très souvent dans les partie molles pré et latéro vertébrales, sous la forme d’abcès froids qui peuvent se développer dans l’espace rétro pharyngé au niveau rachis cervical, dans la gaine du psoas et/ou dans le triangle de Scarpa au niveau du rachis lombaire, dans la fesse ou dans le petit bassin, dans les localisations sacrées. Ces abcès évoluent lentement et peuvent se calcifier et se fistuliser [4]. Au cours des spondylodiscites tuberculeuses, le tableau clinique est peu spécifique [3], entrainant un retard de diagnostic aboutissant ainsi à un volumineux abcès comme c’est le cas de notre patient. Le retard diagnostique fait l’unanimité et se situe entre trois et vingt mois expliquant la fréquence des déficits neurologiques qui sont retrouvés dans des proportions de 20 à 40% [5]. Le long délai de consultation pourrait s’expliqué par le recours au traitement traditionnel en première intention, l’état de pauvreté des patients [5]. L’hypothèse d’un Mal de Pott avait été évoquée, devant le contexte épidémiologique, clinique, radiographique, biologique et évolutif de notre patient. Durant l’étude, nous n’avons pas pu évaluer avec précision le type de lésion vertébrale, l’atteinte des parties molles et péri lésionnelle, ceci par défaut de moyens d’investigations que sont le Scanner et l’IRM non encore disponible au sein de notre structure sanitaire. En effet l’’imagerie par résonnance magnétique est l’examen pour le diagnostic précoce avec une sensibilité de 96% et une spécificité de 92% [2]. Cependant, le Scanner garde l’avantage de mettre en évidence les abcès para vertébral et surtout de guider la ponction biopsie disco-vertébrale [2, 4]. Le diagnostic de certitude de l’origine tuberculeuse est basé sur la mise en évidence *Mycobacterium tuberculosis* dans le liquide de ponction [3], comme ce fut le cas dans notre observation. Le traitement antibacilaire bien conduit pendant une durée suffisante de 12 mois associé à une immobilisation par corset était efficace. L’incision drainage de l’abcès aident à la guérison [3]. En général le traitement chirurgical est indiqué en cas d’atteinte neurologique et en cas d’échec du traitement médical [4], ce qui n’est pas le cas dans notre observation.

## Conclusion

Tout abcès des parties molles de la région rachidienne doit fait évoquer l’origine tuberculeuse. La recherche étiologique doit réunir des arguments anamnestiques, cliniques, et para cliniques. Le traitement quant à lui doit être adéquat afin d’éviter l’évolution vers les complications.

## Conflits d’intérêts

Les auteurs ne déclarent aucun conflit d'intérêt.
